# Trends on the Cellulose-Based Textiles: Raw Materials and Technologies

**DOI:** 10.3389/fbioe.2021.608826

**Published:** 2021-03-29

**Authors:** Catarina Felgueiras, Nuno G. Azoia, Cidália Gonçalves, Miguel Gama, Fernando Dourado

**Affiliations:** ^1^Centre of Biological Engineering, University of Minho, Braga, Portugal; ^2^CeNTI–Centre for Nanotechnology and Smart Materials, Vila Nova de Famalicão, Portugal

**Keywords:** bacterial nanocellulose, fiber, textile, sustainability, cellulose

## Abstract

There is an emerging environmental awareness and social concern regarding the environmental impact of the textile industry, highlighting the growing need for developing green and sustainable approaches throughout this industry’s supply chain. Upstream, due to population growth and the rise in consumption of textile fibers, new sustainable raw materials and processes must be found. Cellulose presents unique structural features, being the most important and available renewable resource for textiles. The physical and chemical modification reactions yielding fibers are of high commercial importance today. Recently developed technologies allow the production of filaments with the strongest tensile performance without dissolution or any other harmful and complex chemical processes. Fibers without solvents are thus on the verge of commercialization. In this review, the technologies for the production of cellulose-based textiles, their surface modification and the recent trends on sustainable cellulose sources, such as bacterial nanocellulose, are discussed. The life cycle assessment of several cellulose fiber production methods is also discussed.

## Introduction

Mankind practices such as the excessive use of non-renewable sources of energy and raw materials, and the unlimited generation of waste in the vast majority of industrial processes, have wide impact on the environment sustainability (Akinsemolu, 2018). Such is the case with the escalating demand for textile products. The demand for textile fibers was 75.5 million tons in 2010 and is expected to increase to 133.5 million tons by 2030 (at a growth rate of 3.1%/year) (Häemmerle, 2011; Eichinger, 2012; Hummel et al., 2015). From the production of raw materials, to spinning, weaving fabrics and dyeing, substantial amounts of water and chemicals are required, including nutrients and pesticides for growing raw materials such as cotton. Downstream, consumers’ use also bare a significant environmental footprint from the consumption of water, energy, and chemicals from washing, drying, and ironing (Sajn, 2019). In 2015, the United Nations set up an agenda aiming to achieve 17 Sustainable Development Goals (SDGs) by 2030, proposing several solutions to improve the economic and social development and providing the essentials to everyone without overwhelming nature (a concept understood as sustainability) (United Nations, 2015). The United Nations Alliance for Sustainable Fashion emerged to ensure the achievement of the SDGs in the fashion industry, throughout the product value chain (United Nations, 2020)^1^.

For a commercial competitive edge, lignocellulose-based materials need to have equal or better characteristics than those of fossil-based ones. Adding functionalities such as conductivity, magnetic properties, bioactivity, water repellency, self-cleaning surface effect, and flame retardance can further improve their competitiveness (Lundahl et al., 2017; Wei et al., 2020). While taking advantage of their nano-scalar dimension, spinning nanocelluloses without solvents provides the possibility of incorporating additives in the filament dope solution while avoiding the use of solvents which are environmentally damaging (Lundahl et al., 2017; Ewulonu et al., 2019; Gao et al., 2019).

The bio-based production of chemicals and microbial technology can also play a major role in this transition toward the future bioeconomy (Choi and Shin, 2020). Various microorganisms already play fundamental roles in our daily life and, if used intelligently, some significant problems associated with the production of green materials can be solved. Indeed, the vast diversity of microorganisms provides huge applications opportunities (Akinsemolu, 2018). The scientific research is still poor comparatively to what these bio-based knowledge areas can offer. In this review we also analyze the opportunities and challenges of using nanocelluloses, from microorganisms and plants, as sources of the most noble natural raw material for the textile industry, cellulose. Production methods used in the textile industry are discussed, such as the methods of Viscose, Lyocell, and Cellulose Acetate, as well as novel processes using Ionic Liquids and solvent-free fibers. Finally, the sustainability of textile fibers and their life cycle assessment (LCA) are analyzed.

## Textile Fibers

### Overview

Fibers are the starting point for all textile products that serve the everyday needs of society. Fibers of short length, called staple fibers, as is the case of most natural fibers, range between 3 and 20 cm in length. A filament is a fiber of indefinite length, being silk the only naturally produced. Most regenerated and synthetic fibers are manufactured as filaments. Distinct methods of drawing, spinning, and twisting, chosen according to the type of fiber, are used to form a continuous strand of yarn (Sinclair, 2015). The filament produced can be used as is, or can be cut into staple fibers. Artificial staple fibers, as the natural ones, must be transformed into yarns. At this stage is very common to blend different fibers, and blends with different combinations of natural and artificial fibers can be found in the market. The textile is then produced. Conventional textiles suit the common decorative or aesthetic usages. Technical textile products can be grouped into various categories, depending on their application, such as industrial, medical, packaging, sports, automotive, construction, aerospatial, geo-textiles, agro-textiles, and protective clothing. Each segment has a huge variety of products made from diversified fibers/raw materials using different manufacturing techniques and equipment (Keller and Giddings, 2020; Rasheed, 2020).

**FIGURE 1:**
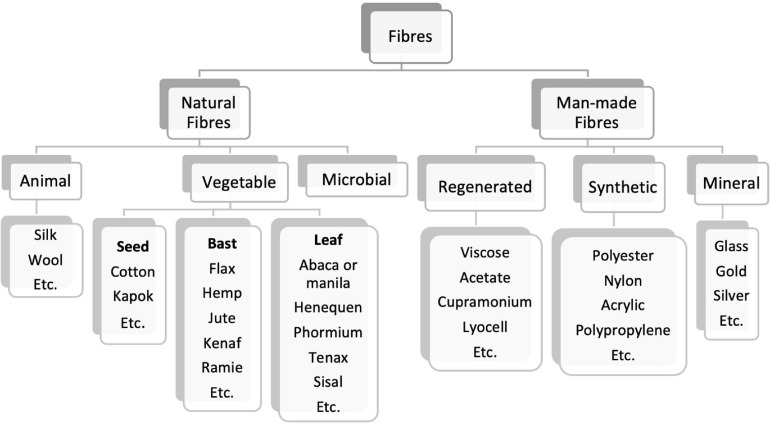
Classification of main natural and man-made fibers [adapted from Murthy (2016), Sinclair (2015)].

Textiles were produced domestically until the 17th century, mostly from vegetable sources using cotton, hemp, and flax but also from animal sources as wool and silk. Then, during the industrial revolution, the manufacture process was mechanized, providing totally new and faster processes (Texcoms, 2019). The fibers produced until the end of the 19th century were all natural (Figure 1). During the 1900s, the production of man-made fibers begun, more specifically regenerated cellulose fibers (RCF) by the Viscose method. This was not the first process for the making of artificial cellulose fibers to be industrialized, but soon became dominant. The synthetic fibers appeared in the textile market only by the 1940’s, made from chemically synthesized polymers (Sinclair, 2015; Murthy, 2016).

Natural fibers, as animal (protein) and vegetable ones, compose up to 40% of the textile fibers manufactured annually in the world. Vegetable fiber (cellulose) is extracted from plants (Yu, 2014). The most abundant natural polymer on planet Earth is cellulose, representing over 30–40% of all terrestrial biomass, with a biosynthesis of 10^11^ tons annually (Levi et al., 2016; Kafy et al., 2017; Akhlaghi et al., 2020). Being biodegradable, renewable, biocompatible, and affordable polymer, cellulose has several other uses such as in paper, cellulose-based plastics, food additives, excipients, coatings, diapers, foams, textiles, and composites (Metsä, 2018; Kim et al., 2019; Havstad, 2020; Stora Enso, 2021). While generally synthesized by plants, it is also produced by some bacteria, fungi, and algae. In plants, cellulose is the main structural constituent of the primary cell wall (Brigham, 2018).

Cotton makes up around 90% of all natural fibers, being the most used one for the making of apparel, home furnishings, and industrial products. It is the main natural fiber crop (Yu, 2014). The use of cellulosic fibers is expected to grow from the current level of 3.7 kg per capita to 5.4 kg by 2030 (Häemmerle, 2011). Cotton fiber contains approximately 90% of cellulose, dried hemp has 40–50% and wood 40–55%, commonly found combined with other substances as lignin and hemicelluloses (Ansell and Mwaikambo, 2009; Wang J. et al., 2019). Additionally to cotton, flax, ramie, jute, kenaf, and sisal are widely used. Cultivation with cotton hybrids will expand and so, the harvest yield from 800 (2010) to 925 kg/ha (2030) will increase its production capacity. However, it will not make up for the disappearance of arable land and growing demand. It is estimated that only 3.1 kg of cotton per capita will be accessible in 2030 (Häemmerle, 2011). This cellulose gap provide new opportunities for man-made cellulosic fibers (MMCF). The gradual substitution of cotton by pulp-based fibers is also required from an environmental point of view. Nowadays, new natural fibers, mainly vegetable ones, are being utilized as kapok, pineapple, and apocynum (Yu, 2014).

Synthetic fibers have dominated the market since mid 1990s, overtaking cotton. These are made from organic synthetic high-molecular mass compounds and are produced synthetically from petroleum-based raw materials. They represented up to 63% of the global fiber production in 2019. The most used synthetic fiber was polyester, with a market share of around 52% of total global fiber manufacture. Cotton was second, with 23% (Textile Exchanges, 2020). Currently, fibers have a cost of 1.17 €/kg for cotton, 1.31 €/kg for Viscose, and 0.86 €/kg for polyester (Emerging Textiles, 2020)^2^.

### Novel Sources of Cellulose and Nanocelluloses

In recent years, several nanocelluloses (NC), from microbial and plant sources, have been tested as a source of textile fibers. The interest in NCs is essentially focused on taking advantage of their higher crystallinity, since they promote great mechanical resistance (Nunes, 2014). NC is not a single material type but rather a family of materials with very distinct features, mostly due to different sources and preparation methods (Clemons, 2016).

#### Bacterial Nanocellulose

**FIGURE 2:**
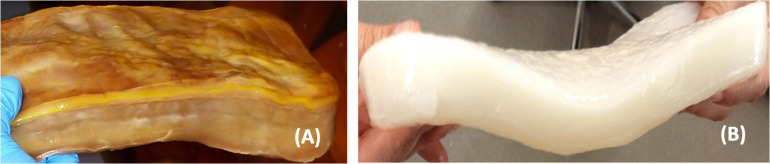
BNC membrane before **(A)** and after **(B)** purification.

An alternative to wood/plant cellulose is bacterial nanocellulose (BNC) (Figure 2), a homopolysaccharide extruded by Gram-negative species of the genera *Komagataeibacter*, *Acetobacter*, *Rhizobium*, *Agrobacterium*, *Pseudomonas*, *Salmonella*, *Alcaligenes*, and *Sarcina*, the only Gram-positive bacterial genus (Jonas and Farah, 1998; Dourado et al., 2016b). Different bacteria produce cellulose with distinct morphology, structure, properties, and yields (Wang J. et al., 2019). To obtain high yields of BNC, it is necessary to use the highest cellulose producer species, such as *Komagataeibacter xylinus* (Dourado et al., 2016). In addition to using high BNC producers, other approaches toward improving the production of BNC include the use of advanced reactors, complex culture media or even the development of cell-free enzyme systems (Ul-Islam et al., 2020).

The BNC biosynthesis was first observed in kombucha, a fermented beverage produced by a symbiotic colony of bacteria and yeast, where a cellulose film is weaved on the culture media-air interface. It was first reported in Brown (1886), who identified a film with a structure chemically equivalent to that of plant cellulose. BNC consists of <100 nm ribbon-shaped fibrils, with 7–8 nm wide nanofibrils randomly aggregated into bundles, without lignin or hemicellulose (Gorgieva and Trček, 2019).

Bacterial nanocellulose has a chemical structure identical to that of plant cellulose. Linear homopolymer of glucose monomers are linked by β-(1→4) glycosidic linkage with the chemical formula (C6H10O5)*n*. Nevertheless, it has different macromolecular structure and properties (Ullah et al., 2019). The polymerization degrees are within 2000–6000 for BNC and 13,000–14,000 for plant cellulose, decreasing during pulping and purification. The unbranched chains of cellulose are held together through strong intra- and intermolecular hydrogen bonds to make the elementary fibers and the supramolecular structure (Choi and Shin, 2020; Fang et al., 2020). This structure possesses exclusive features, such as high mechanical strength, water-holding capacity, dimensional stability, crystallinity, biocompatibility, and biodegradability (Picheth et al., 2017). Given these features, numerous applications of BNC have been studied: in the biomedical field as a wound dressing, for tissue regeneration/substitution, drug delivery systems, biosensors and cancer diagnosis; in the textile and paper industries for fiber composites and coatings; in the food and cosmetic industries as an emulsifier and viscosifier (Klemm et al., 2005; Chawla et al., 2009; Müller et al., 2013; Nimeskern et al., 2013; Lee et al., 2014; Shi et al., 2014; Rajwade et al., 2015; Ul-Islam et al., 2019; Amorim et al., 2020; Anton-Sales et al., 2020; Cabañas-Romero et al., 2020; Farooq et al., 2020; Liu et al., 2020; Nanollose, 2020). The main application is still as a food item known as *nata de coco*, mostly produced and consumed in Asian countries (Dourado et al., 2016a).

Bacterial nanocellulose can be produced through static or agitated cultures (Wang J. et al., 2019), most studies being carried out using the former (Zywicka et al., 2015). In this method, BNC is produced in containers filled with nutrients and incubated for 1–14 days, at 28–30°C and pH 4–7 (Wang J. et al., 2019). The efficiency of BNC production in stationary cultures is strongly connected with the air–liquid surface area (given that *Komagataeibacter* strains are mandatory aerobes), where it is produced as a hydrogel sheet containing around 99% of water (Wang J. et al., 2019).

To achieve industrial scale production, alternative fermentation technologies have been studied, using specific fermentation media and overproducing mutant strains include agitated and air-lift bioreactors, membrane reactors and horizontal bioreactors. The idea behind is that the agitated/shaking culture facilitates the oxygen delivery to bacteria during cultivation (Wang J. et al., 2019). Agitation and aeration leads to the formation of fibrous suspensions, which limits BNC applications and favors cellulose-negative mutants to control the population (limiting the cellulose yield), in addition to requiring high stirring power (as the viscosity of the suspension is too high) (Czaja et al., 2004; Huang et al., 2014; Rodrigues et al., 2019; Wang J. et al., 2019).

The textile fibers production amounts to 105.6 million tons per year, worldwide (Garside, 2019). The current BNC global production is far below this magnitude and confined to small scale production units (Dourado et al., 2016a; Phisalaphong et al., 2016; Piadozo, 2016). BNC’s production costs are generally considered very high, preventing the scale of production from increasing to industrial levels. Indeed, *nata de coco* costs around 50–80$US, per kg (dry weight equivalent). The high cost of the fermentation medium has been pointed out as one main difficulty for the large-scale production of BNC. This may be overcome by using residues from the food industry as a low cost medium, which in addition to reducing the cost of production also helps to solve environmental problems related to waste disposal (Ul-Islam et al., 2020). However, according to other authors, the impact of the nutrients on the final cost is not so relevant, and the use of residues from the food industry may lead to more heavily charged wastewaters, hence more expensive to treat (Dourado et al., 2016a). Another big difficulty lies on the low BNC yield. In order to increase the yield of BNC produced, better strains are required, either isolated from nature or modified by genetic engineering techniques (Zhong, 2020). It must also be recognized that a comprehensive assessment of the very large scale industrialization potential of BNC remains to be accomplished, therefore its potential as a sustainable alternative for the textile industry cannot be fully ascertained at present (Choi and Shin, 2020).

#### Plant Nanocelluloses

**FIGURE 3:**
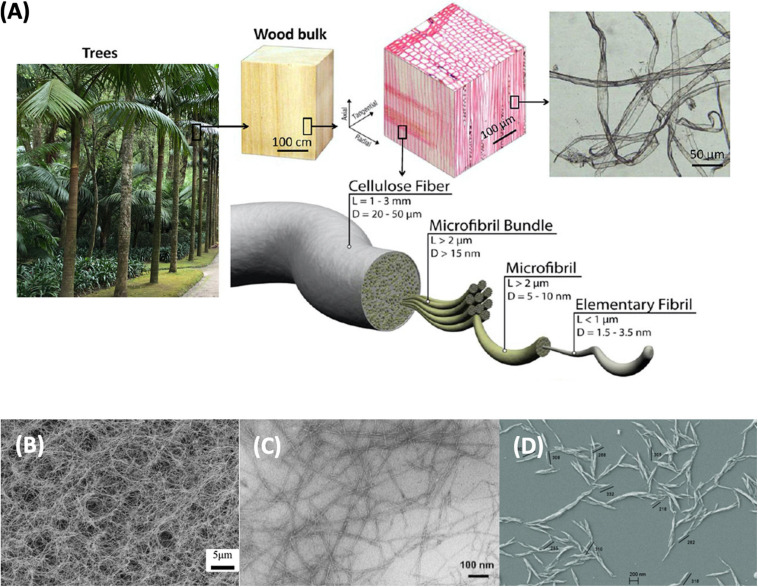
**(A)** Hierarchical structure of wood fibers: Reprinted (adapted) with permission from Zhu et al. (2013). Copyright (2013) American Chemical Society. Comparison microscopic image of; **(B)** bacterial nanocellulose (Li et al., 2017): Published by The Royal Society of Chemistry, **(C)** nanofibrillated cellulose: Reprinted (adapted) with permission from Saito et al. (2007). Copyright (2007) American Chemical Society and, **(D)** nanocrystalline cellulose (Anžlovar et al., 2018).

The hydroxyl groups on one cellulose chain bond with the other to develop rigid and stable molecules, giving the plant stiffness and strength. The hydrogen bonding between cellulose chains makes it insoluble in water (Thomas et al., 2020). Fibrils are formed by joining cellulose molecules together. In turn, fibrils agglomerate into bundles, which the plant uses to form the cell wall combined with hemicelluloses and lignin (Figure 3; Lundahl, 2018). The cellulose fibrils morphology demonstrates a mesh-like structure (Thomas et al., 2020).

Nanocelluloses (NC) can be produced by top-down approaches, i.e., the dismantling of fibers by chemical, enzymatic, or mechanical methods in case of plant NC, or by bottom-up routes for BNC (Charreau et al., 2020). Plant NC is generally categorized in cellulose nanofibers (CNFs) and cellulose nanocrystals (CNCs) (Amorim et al., 2020). The final chemical and physical properties of NC depend directly on the source and manufacture conditions (Kargarzadeh et al., 2017). NCs have characteristics such as high strength and stiffness, low density, biodegradability, high surface area, and low thermal expansion, which led to much research and innovation during the last two decades. Both CNC and CNF have applications as composite materials, paper and board industry, adsorbent products, food and beverages, paints and coatings, adhesives, packaging, oil and gas, electronics, and medical, pharmaceutical, and cosmetic product (Charreau et al., 2020).

Different raw materials can be used to obtain NCs such as coconut husk fiber, mengkuang leaves (*Pandanus tectorius*), cotton, *Agave tequilana*, barley wastes, tomato peels, garlic straw residues, forest residues, corncob residue, *Gigantochloa scortechinni* bamboo culms, industrial waste cotton, cassava root bagasse and peelings, sugar palm fibers (*Arenga pinnata*), corn straw, and sago seed shells (Ventura-Cruz and Tecante, 2019). The raw material needs to be pre-treated to remove lignin and hemicellulose by milling, pulping and bleaching. CNCs, also named cellulose whiskers, nanowhiskers, or nanorods, are manufactured by transverse cleavage of cellulose using strong acids such as sulfuric and hydrochloric under defined conditions of temperature, agitation, and time (Clemons, 2016; Charreau et al., 2020). CNC have a nanosized distribution: a diameter of 4–55 and 90–400 nm in length (Zinge and Kandasubramanian, 2020). After acid hydrolysis, CNC is obtained following washing, filtration/centrifugation, and dialysis, to take out the remaining acid (Charreau et al., 2020). Due to CNC higher crystalline structure, it has less flexibility than CNF (Zinge and Kandasubramanian, 2020). Currently, CNC is produced in commercial quantities of 2–260 ton/year (Charreau et al., 2020).

Cellulose nanofibers, also called cellulose nanofibrils or nanofibrillated cellulose, are obtained by mechanical disintegration (Amorim et al., 2020). CNF are cellulose structures of high aspect ratio bearing crystalline and amorphous regions. Although CNF isolation is associated with mechanical destructuring methods (pressure, cavitation, shear, and impact forces), the high energy consumption needed has led to the integration of pre-treatments to facilitate further fibrillation (Charreau et al., 2020). A common pre-treatment uses a 2,2,6,6-tetramethylpiperidine-1-oxy radical (TEMPO) catalyst to mediate the oxidation of native celluloses, that lowers the energy needed to fibrillate (Clemons, 2016). The product obtained is a translucent firm gel. CNF can be applied in absorbent materials, for the reinforcement of composites, as a rheology modifier agents and, mainly in papermaking, namely of paperboard, tissue, deodorant sheets, and cosmetics sheets. CNF is produced in commercial quantities of 24-560 ton/year (Charreau et al., 2020).

## Technologies for the Production of Cellulose Textile Fibers

### Regenerated Cellulosic Fibers

Regenerated cellulose fibers are obtained by dissolving cellulose, pure or derivatized, from wood pulp or plant fibers. As the length of wood pulp fibers is too small for textile use, they need to be processed using continuous spinning and a regenerating technology (Navard, 2013). In the derivatization step the structure of the starting cellulose is changed, forming an intermediate compound such as sodium xanthate or acetate derivatives. Then these intermediate compounds are prepared and dissolved, along with the regeneration of the fiber (Woodings, 2001). The improvement of cellulose dissolution is a prevailing goal. The cellulose structure is transformed in different ways depending on the type of solvent and treatment conditions. Cellulose solvents can be divided into two groups: non-derivatizing–whereby the polymer is dissolved by intermolecular interactions; derivatizing–where the dissolution process is combined with the formation of unstable ether, ester or acetal derivatives. Both groups include aqueous and non-aqueous solvents (Heinze and Koschella, 2005). Presently, the most used industrial methods for dissolving cellulose pulp are the Viscose, Cuprammonium, and Lyocell methods.

Regenerated cellulose was the first man-made fiber utilized in the textile industry, in the beginning of its development, in the early twentieth century. These fibers have a smooth and lustrous silk-like aspect, combined with the outstanding water absorption capacity of cotton. In 1924 the generic name Rayon was accepted by the U.S. Department of Commerce and some industrial corporations to label RCF that include Viscose, Acetate, Lyocell, Modal, and Cupro (Chen, 2015; Textile Exchanges, 2020).

With an annual production volume of around 7.1 million tons in 2019, the global production volume of RCF has more than double since 1990. RCF have a market share of about 6.4% of the total fiber production volume and is expected to increase in the coming years. Viscose is the dominant RCF with a market share of around 79% of all RCF and a production volume of around 5.63 million tons in 2019. Acetate has a market share of around 13% of all RCF with a manufacture of roughly 0.95 million tons in 2019 but it is largely utilized for non-textile applications. Lyocell ranks third most utilized RCF after Viscose and Acetate in 2019. It had a market share of around 4.3% and a production volume of 0.3 million tons, being this fiber expected to grow faster than the others RCF. Using a production process quite similar to that of Viscose, Modal fibers had a market share of around 2.8% of the total RCF market in 2019 with a production of around 0.2 million tons. Cupro has a market share of less than 1% of the total RCF market. There is only one provider of Cupro, manufacturing 17,000 tons in 2019 (Textile Exchanges, 2020).

Regenerated cellulose fibers are being used in the most diverse materials, from sportswear to health care textiles, alone or combined with other natural or synthetic fibers, thanks to their characteristic properties as tensile strength and smoothness (Karthik and Rathinamoorthy, 2017).

#### Viscose Rayon

The Viscose process is the world’s most widely used method for producing RCF. The first patent on the Viscose method was granted to Cross and Bevan in 1893. Over the past 100 years, this process underwent many alterations, although the basic chemistry is still the same, which allowed Viscose to become one of the most widely used regenerated fibers (Wilkes, 2001; Thakur et al., 2017).

**FIGURE 4:**
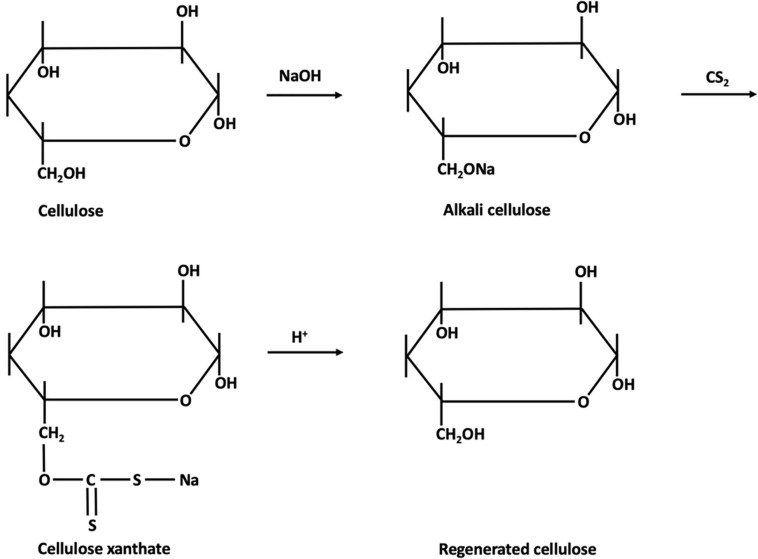
Mechanism of Viscose fiber production [addapted from McKeen (2017), Rodgers and Waddell (2013)].

**FIGURE 5:**
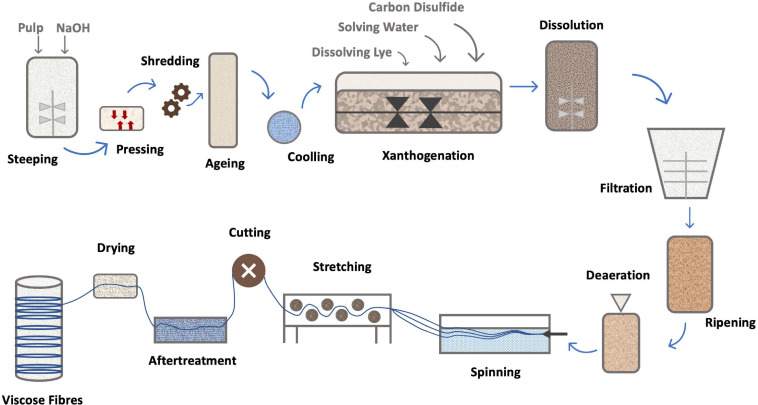
Schematics of Viscose fiber production [addapted from Alagirusamy and Das (2015), Sayyed et al. (2019)].

The process, shown in Figures 4, 5, consists in suspending the pulp in NaOH and, after steeping for a specified period of time, shredding and aging. The viscosity of the pulp depends on the aging time. The aged pulp is then treated using carbon disulfide (CS_2_) to form the orange-colored cellulose xanthate. Lastly, this derivative is dissolved in NaOH with a lower concentration, the starting stage of Viscose formation (Shaikh et al., 2012). The polymer is finally precipitated in acid, for simultaneous neutralization and regeneration of the cellulose using a wet-spinning equipment. Then, several steps of washing and drawing yield a regenerated fiber of pure cellulose (Olsson and Westman, 2013; Chen, 2015). Currently, carbon disulfide can be reused up to 70%, the remaining being converted into sulfuric acid (H_2_SO_4_), which is also recovered (Rana et al., 2014).

Despite being made from wood, the fiber production by Viscose process is known to cause significant environmental problems due to the high usage of chemicals, such as sodium hydroxide, producing sodium sulfate as a by-product. The life cycle of the Viscose process encompasses other impacts, associated to how reagents are produced and recycled, as well as the consumption of energy, the use of fossil fuel and deforestation (Shen et al., 2010).

Recently, the company Nanollose announced the development of a Viscose making process using BNC. This method transforms BNC into Nullarbor^TM^ Tree-Free Viscose fibers using manufacturing equipment compatible with those currently used by the industry (Jinzarli et al., 2019; Nanollose, 2020)^3^.

#### Lyocell Rayon

The direct dissolution of cellulose (without derivatization) has been the object of research for many years. This process may also ease the manufacture of regenerated cellulose by omitting several steps (Perepelkin, 2007; Olsson and Westman, 2013). The technology of direct dissolution of cellulose is a simpler process, reducing the use of chemicals by ten times in comparison to the Viscose process. Also, a direct solvent is easier to recycle, since no byproducts are formed, resulting in a more environmentally friendly process (Olsson and Westman, 2013). However, the Lyocell production costs are higher than the Viscose, due to the high cost of solvent and the use of high temperatures for cellulose dissolution (Alam and Christopher, 2017).

##### History of Lyocell

Lyocell is the first successful technology of cellulosic fibers by direct dissolution, resulting in fibers with exceptional properties, making it a serious competitor to the Viscose process, since it is more environmentally friendly (White, 2001; Peng et al., 2017). Lyocell fiber has higher tenacity (especially wet tenacity), higher modulus, lower shrinkage, better thermal stability, higher crystallinity, and greater degree of orientation, than Viscose (Edgar and Zhang, 2020). The Lyocell fiber market is estimated to grow from 760 million € in 2016 to over 1.35 billion € by 2024 (Pulidindi and Chakraborty, 2016).

A patent describing the process of cellulose dissolution using *N*-methylmorpholine-*N*-oxide (NMMO) solvent was filled by Mcorsley (1981), In 1992, in Mobile Alabama, United States, the company Courtaulds, achieved the full commercial production with the trade name TENCEL^®^ (Chen, 2015). Lenzing, an Austrian company, started the production of Lyocell fiber in 1990 with their first pilot plant. The full-scale production plant came into operation in 1997 and the fiber was called Lenzing Lyocell^®^. In 2004, Lenzing purchased the TENCEL^®^ Group (Perepelkin, 2007). Today, Lenzing is the world’s biggest Lyocell fiber producer (Rana et al., 2014). Other companies have emerged with new brand names for fibers obtained using this technology, as Alceru (TITK Rudolstadt), Newell (AkzoNobel), Acelon (Greencell), and Excel (Grasim) (Sayyed et al., 2019).

##### The solvent and phase diagram

*N*-methylmorpholine-*N*-oxide, commonly known as NMMO, is the most used of all non-derivatizing cellulose solvents, due to its capacity to directly dissolve high concentrations of cellulose, while preserving the chemical properties of the polymer. The possibility of recycling up to 99.7% of the solvent, makes the process economically viable and more environmentally friendly (White, 2001; Olsson and Westman, 2013).

**FIGURE 6:**
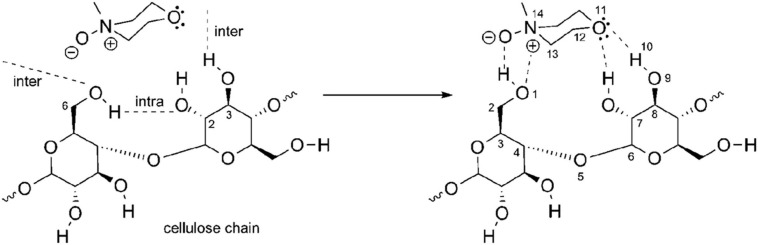
*N*-methylmorpholine-*N*-oxide–cellulose dissolution mechanism: Reprinted with permission from Pinkert et al. (2010). Copyright (2010) American Chemical Society.

*N*-methylmorpholine-*N*-oxide is completely soluble in water and very hygroscopic. The polarity of the N–O bond also results in a great ability to form hydrogen bonds. There are three types of NMMO: anhydrous NMMO, monohydrate NMMO (NMMO⋅H_2_O) with 13.3% (w/w) H_2_O, and disesquihydrate NMMO (NMMO⋅2.5 H_2_O) with 28% (w/w) H_2_O (Wikandari et al., 2016). NMMO’s oxygen can form two hydrogen bonds with hydroxyl groups from water or cellulose (Figure 6), the competition between water and cellulose for these hydrogen bonds, being the central feature of this dissolution process (Maia et al., 1981).

Since NMMO is thermally unstable, the dissolution of cellulose in NMMO at high temperatures is accomplished in well-controlled environment. NMMO degrades at temperatures higher than 120°C. Due to its reactivity, stabilizers should be added at the start of the dissolution process, most commonly propyl gallate (Rosenau et al., 2002).

The degradation of NMMO and following side reactions may include *N*-methylmorpholine, morpholine, and formaldehyde (Olsson and Westman, 2013). Some reactions are started by transition metal ions such as iron and copper and that’s why these should not be used in this process (Klemm et al., 2005). Mechanical energy facilitates the rupture of cellulose-cellulose intermolecular bonds, favoring the interaction with NMMO. Furthermore, the apparent viscosity decline with shear stress due to cellulose alignment, as anticipated for non-Newtonian polymer solutions. Cellulose concentration and degree of polymerization (DP) also influence the viscosity (Olsson and Westman, 2013).

**FIGURE 7:**
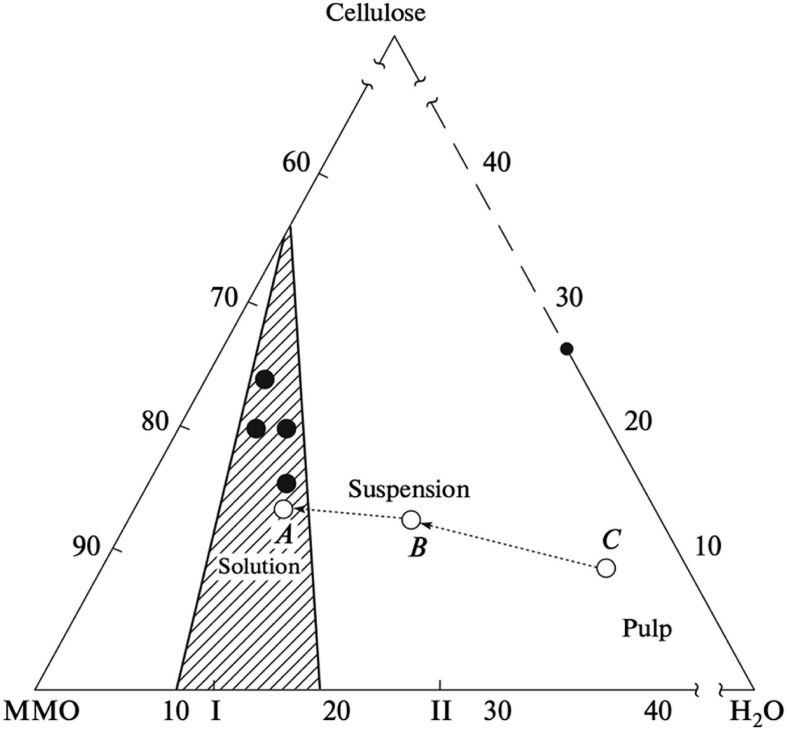
Schematic phase diagram of the cellulose–MMO–H_2_O system: (line CBA) variation in the composition of the system during cellulose dissolution via the traditional MMO process; (closed circles) composition of the system during cellulose dissolution via the solid-phase MMO process; (I) MMO monohydrate and (II) MMO 2.5-hydrate (Golova et al., 2013).

The NMMO-water-cellulose phase diagram (Figure 7), shows the relative amounts of H_2_O and NMMO required for cellulose dissolution to occur. NMMO–water mixtures are direct solvents for cellulose in its monohydrate state. With a higher amount of water, cellulose will not dissolve, since a competition for NMMO takes place between cellulose and water, the interaction with the latter being favored. Thus, NMMO only links with cellulose when there is shortage of water molecules (Biganska and Navard, 2003). The process (described in more detail in the next section) starts with using a high water content, to induce the swelling of the cellulose fibers. An homogeneous pulp is produced containing, e.g., 35% of H_2_O, 9% cellulose, and 56% NMMO (point C in Figure 7). Then, the excess of water is removed under vacuum (point B). After further water removal, dissolution occurs at point A with a 14% cellulose, 10% H_2_O, and 76% NMMO (Golova et al., 2010).

##### Dissolution process

**FIGURE 8:**
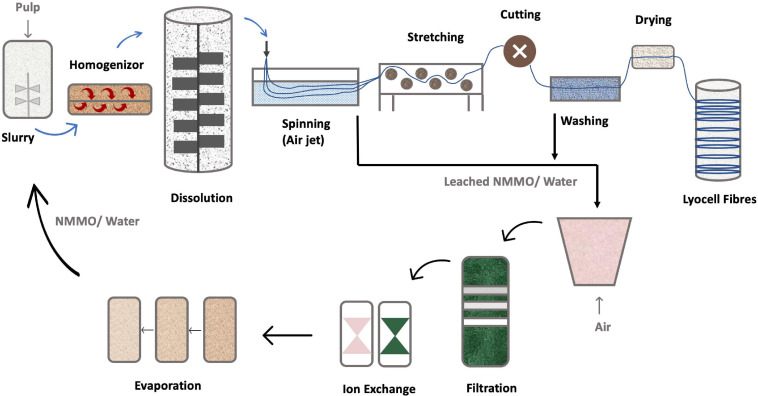
NMMO based lyocell process [addapted from Jiang et al. (2020), Sayyed et al. (2019)].

The first industrial system used to dissolve cellulose with NMMO (Figure 8), involved the initial cellulose swelling. A cellulose pulp must first be milled into small particles to improve the surface contact area with NMMO. Then it is mixed with NMMO containing 20–30% of water (Zhang S. et al., 2018). The amount of pulp fed to the mixer has to be quantified, to control the cellulose content. Mixing is done at 70–90°C, with high-speed refiners, to further break down the pulp, improving the solvent wetting. The resulting swollen pulp is then heated to 90–120°C, under vacuum, to remove excess water, yielding a clear dark amber-colored solution, to a final cellulose concentration within 10–18% (m/m) (White, 2001)The use of low pressure reduces the temperature required for water evaporation, so the NMMO does not undergo exothermic degradation (White, 2001). After dissolution, impurities such as undissolved pulp fibers are filtered. The mixture is then extruded and spun through an air gap into a spin bath with a polar liquid, like water or alcohol. These coagulation agents are miscible with NMMO, removing it from the cellulose solution (Biganska and Navard, 2011). This process is called dry-jet wet-spinning and consist of thousands of small holes through which the solution is extruded into fibers (White, 2001). Various other technologies are used in fiber spinning dope such as kettle-type dissolution, twin-screw extruder dissolution, vacuum mixed propulsive dissolution and vacuum membrane propulsive dissolution technology. The first one is intermittent, the others are continuous. Currently, the most used technology in industrial manufacturing is continuous vacuum film propulsive dissolution technology (Jiang et al., 2020).

To facilitate the cellulose dissolution process, various pre-treatment methods have been researched such as enzyme treatment, to enhance reactivity, microwave heating process to reduce crystallinity, mechanical blending technique, enzymatic peeling treatment, ball or vibration milling, steam explosion, and electron beam irradiation. However, industrially, most of the these are technically and economically non-viable (Sayyed et al., 2020). Pre-swelling at temperatures from 30 to 75°C lowers the pulp’s crystallinity and forms a homogeneous solution with lower time and power consumption during the dissolution process. This makes Lyocell process more energy-efficient and sustainable (Sayyed et al., 2020).

The DP of cellulose pulp influence the mechanical strength of Lyocell fiber. The higher DP, the superior the mechanical strength; yet, a very high DP may result in a poorer solubility and an increase in the viscosity of spinning dope. The balance between DP and the solubility of dissolving pulp has a substantial impact on the spinning process and the mechanical properties of fiber (Jiang et al., 2020). Due to the difference in the DP between BNC and plant cellulose, the use of the same concentration may yield different results, given the large difference in viscosity. For example, a 6% (m/m) solution of BNC (DP 2000) has a viscosity 10^3^ Pa.s, which in the case of wood cellulose (DP of 600) is achieved only at 18% (m/m) (Makarov et al., 2019).

Bacterial nanocellulose Lyocell fibers have been produced by Gao et al. (2011). In their work, water was added to NMMO at 13.3% m/m and the mixture was heated at 90°C for 30 min. Then BNC powder was added (7% m/m) and the mixture was heated at 80°C for 12 h. The solution was extruded and BNC filaments were formed. The regenerated BNC fibers had a cellulose II crystalline structure, lower degree of crystallinity, smaller crystalline sizes, and better thermal stability than native BNC. This regenerated BNC fibers had a tensile strength of 0.5–1.5 cN/dtex and extension at break of 3–8% (Gao et al., 2011).

A different process for Lyocell fiber production, using the NMMO-water-cellulose solid-phase system at a concentration of cellulose under 25% (m/m), was developed at the All-Russian Scientific-Research Institute of Polymer Fibers (Golova et al., 1992; Golova, 1996). The method uses high-melting hydrate forms of NMMO, which better dissolves cellulose, substantially accelerating the process. The process starts with a solid-phase activation of cellulose by NMMO with a water content of 8% (m/m) or less. Cellulose dissolution occurs under triaxial compression, shearing, and forced plastic flow. Then, under temperature (105°C) and shear rate, the cellulose dissolves into a highly homogeneous and concentrated solution (Golova et al., 2013). This method has been successfully used with BNC fibers with a DP of ∼1500 and a solid-phase activation of BNC and NMMO (water < 10%); the mixture was heated at 120°C until a fluid solution was obtained (Makarov et al., 2019). The authors claim that the (mechanochemical) activation is mandatory for a homogeneous solutions to be obtained, as no swelling occurs. Concentrations of up to 8% of BNC were used, 6% being optimal. The fibers were spun by dry-jet wet-spinning into a water coagulation bath. The mechanical properties of the obtained fibers are shown in Table 1, along with other synthetic, natural and regenerated (man-made) cellulosic and organic synthetic fibers. The mechanical properties of Lyocell produced with BNC are similar with data for Viscose and Lyocell fibers (Makarov et al., 2019, 2020).

**TABLE 1:** Mechanical properties of different fibers.

	d (μm)	Young’s Modulus (GPa)	Tensile Strength (MPa)	Elongation (%)	References
BNC Lyocell	10.3–15.4	8.6–10.1	420–495	5.5–6.5	Makarov et al., 2019
Ioncell	–	30	700–800	–	Sashina, 2019
Lyocell	9	22–31	472–624	6.8–13.7	Gindl et al., 2008; Manian et al., 2018
Viscose	10.5	9.3–11.6	220–340	8–23.5	Gindl et al., 2008; Manian et al., 2018
Cotton	10–27	8	600	7	Bunsell, 2018
Wool	15–40	2	170	35	Bunsell, 2018
Flax	15–20	65	650	1–3	Bunsell, 2018
Silk	12	8	400	25	Bunsell, 2018
Hemp	45	50	500	1–2	Bunsell, 2018
Jute	69	35	350	2.5	Bunsell, 2018
Polyamide 66 (Nylon 66)	20	<5	1000	20	Bunsell, 2018
Polyester (PET)	15	15	800	15	Bunsell, 2018
Kevlar 49	12	135	3000	4.5	Bunsell, 2018

Nonetheless, the Lyocell process has some limitations related to NMMO’s intrinsic properties. The N–O moiety blocks the application of redox-active agents, whereas the cyclic ether structure is susceptible to thermal runaway reactions, requiring stabilizers. The side reactions and considerable byproduct formation can cause the degradation of cellulose, a temporary or permanent discoloration of the spun fibers, a drop in product performance, a relevant decomposition of NMMO and higher consumption of stabilizers. So, alternative direct solvents for cellulose dissolution would be highly attractive for environmental and economic reasons (Rosenau et al., 2001; Hummel et al., 2015).

#### Cellulose Acetate

**FIGURE 9:**
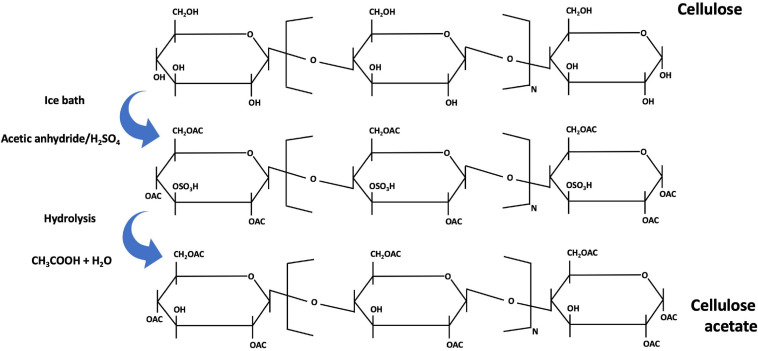
Mechanism of the cellulose acetate preparation [addapted from Sayyed et al. (2019), Silva et al. (2017)].

Although not so widely used, another method of producing fibers is available whereby cellulose acetate is obtained. Cellulose acetate is the acetate ester of cellulose. It was first manufactured at commercial scale by Celanese in 1923 (Sayyed et al., 2019) by reacting a cellulose pulp with acetic anhydride, to form acetate flakes (Figure 9). Then, these flakes are dissolved in a solvent and filtered to produce the spinning cellulose dope solution (Ertas and Uyar, 2017). The cellulose dissolution with acetic acid and acetic anhydride is done in the presence of sulfuric acid. Partial hydrolysis of cellulose acetate in then performed in a controlled manner, to remove the sulfate and a sufficient number of acetate groups, to yield a product with wanted properties. The cellulose dope solution is then extruded through a spinneret and the yarns are produced by solvent evaporation. This process for producing acetate fiber is made using the dry-spinning method (Sayyed et al., 2019), and is mainly used to produce cigarette filters but also for drug delivery and nanofibers. These cellulose acetate fibers have limited use in the textile industry because of their poor strength, poor abrasion resistance and poor thermal retention (Watabe et al., 2018).

#### Ionic Liquids

Research on the use of Ionic liquids (ILs) for the direct dissolution of cellulose, first used in 2002, has shown promising results. A new class of next-generation RCF named Ioncell have recently been developed utilizing a novel IL solvent 1,5-diazabicyclo[4.3.0]non-5-enium acetate ([DBNH] [OAc]) (Michud et al., 2016). Ioncell fibers have been shown to exhibit better mechanical properties than all previously known ones, namely, their tensile strength reaches 0.7–0.8 GPa and elastic modulus of 30 GPa (Wanasekara et al., 2016; Sashina, 2019). Several garments have been produced and commercial production is expected by 2025 (Ioncell, 2020)^4^. The IL process is carried out using the Lyocell technology since the cellulose solution is spun according to the NMMO-based Lyocell method, in a dry-jet wet-spinning process where the filament go through an air gap and coagulate in a bath where the regenerated cellulosic fibers are formed (Sayyed et al., 2019).

Ionic liquids are liquids made-up of cations and anions, thus they can be designated as salts in the liquid state (Singh and Savoy, 2020). Overall, the dissolution process of cellulose in ILs is still not fully clarified (Hermanutz et al., 2019). The ILs ability to dissolve cellulose is due to small hydrogen bond accepting anions that can compete with the hydrogen bonding between the cellulose chains and cations that help increase the solubility (Andersson, 2018). ILs dissolves cellulose with no prior derivatization in concentrations of up to 300 g.L^–1^ and offer a potentially more environmentally friendly alternative to traditional processes (Hina et al., 2015; Wanasekara et al., 2016). These solvents are thermostable up to 300°C with a melting point up to 100°C. ILs practically do not have vapor pressure, and therefore do not pollute the atmosphere (Sashina, 2019). Due to their wide range of properties, they have been increasingly used in various fields of study such as biochemistry, engineering, physics, etc. as a green solvent. The properties of ILs can be modified depending on their application by altering the combination of cations and anions (Singh and Savoy, 2020).

Ionic liquids usually comprise imidazolium, pyridinium, or organic ammonium cations and anions such as chloride, bromide, or more complex structures such as hexafluorophosphate, trifluoromethyl sulfonate, bis(trifluoromethylsulfonyl)imide, and methylimidazolium chloride ([Amim]Cl) (Sayyed et al., 2019). ILs have been investigated either to dissolve or to create appropriate support for the functionalization of cellulose. For cellulose solubility, the counter anions with halide, such as the imidazolium type, have the best performance. One disadvantage with ILs with halide anions is their relatively high viscosities, which brings processing difficulties during dissolution (Isik et al., 2014). These ILs demonstrate good dissolution characteristics, allowing the production of cellulose dopes in concentration ranges that shows good spinnability (Sayyed et al., 2019). For industrial use, any IL selected for cellulose dissolution and processing has to match specific criteria for an economic process development: the IL should be easy to produce, recyclable in high amount (>99.5%), possess the lowest possible toxicity, should have literally no vapor pressure, a low melting point, a low propensity to side reactions and degeneration, have high dissolution capability for different pulp sources (Andersson, 2018; Hermanutz et al., 2019). To obtain concentrated cellulose solutions suitable for spinning, the researchers have tried to use ILs based on imidazolium, pyridinium and ammonium cations (Sashina, 2019). Currently, nearly thousand ionic liquids are described in the literature (Meksi and Moussa, 2017; Wang H. et al., 2019; Zhang et al., 2019; Yang et al., 2020).

### Filaments Without Solvents

Regenerated cellulose fibers have beneficial characteristics from both synthetic and natural fibers: they have uniform morphological, mechanical, and physical properties, as synthetic fibers. Also, they bear biodegradability, CO_2_ neutrality, and low density of natural fibers. Still, their mechanical properties are lower than those of CNF (Hooshmand et al., 2015). To preserve the characteristics of CNF, several studies have been made to elaborate a system of filament production without solvents. Spinning CNF requires lower energy use and no harmful chemicals are used (Lundahl et al., 2017). Instead of dissolution, CNF are dispersed in water and spun into air or reusable organic solvents. This process was announced in 2011 and has since then progressed in the improvement of the fibers’ mechanical properties and process scale-up (Iwamoto et al., 2011; Lundahl, 2018).

An isolated cellulose crystallite has a Young’s modulus of up to 160 GPa and tensile strength of 6–7 GPa, exceeding those of carbon and Kevlar fiber in its longitudinal direction, while in the transverse direction a value of 8–57 GPa is observed (Wang et al., 2017). Thus, the mechanical performance of cellulose can be optimized when the crystallites are well aligned, a goal that can be accomplished by spinning (Lundahl, 2018). Wet and dry-spinning have to date been applied on CNF at laboratory scale, using a syringe pump, extruder, capillary rheometer, or 3D printer (Lundahl, 2018).

**FIGURE 10:**
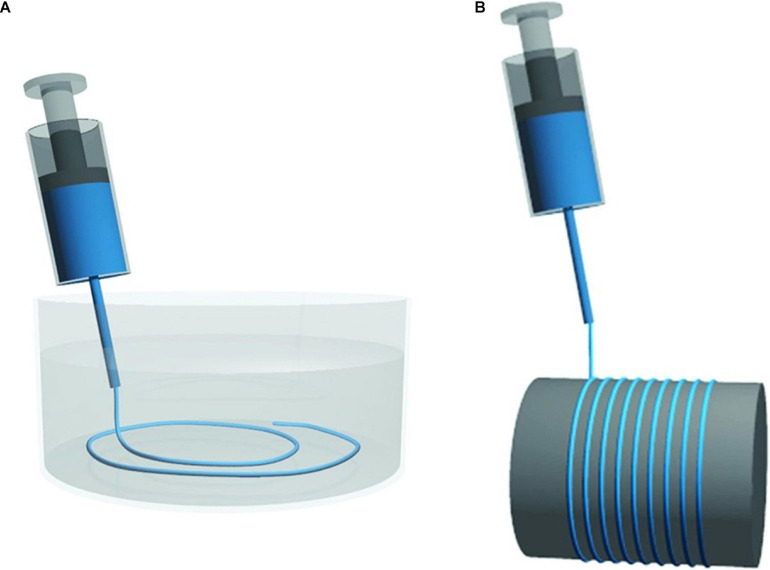
Schematic illustrations of simplified systems for **(A)** wet-spinning and **(B)** dry-spinning. Reprinted (adapted) with permission from Lundahl (2018). Copyright (2018) American Chemical Society.

In the production of filaments, TEMPO oxidized CNF is used (Iwamoto et al., 2011) and extruded through a spinneret in a wet or dry-spinning process (Figure 10). In wet-spinning, a coagulation bath with acetone, water, ethanol, or CaCl_2_ solution is used. The characteristics for the coagulants are miscibility with water, moderate polarity and hydrogen bonding ability (Iwamoto et al., 2011; Walther et al., 2011; Kim et al., 2019). The coagulant bath rapidly induces the generation of “a skin” on the surface of the CNF extrudate, stabilizing it against interfiber aggregation, to allow the formation of distinct macrofibers (Walther et al., 2011). In dry-spinning, the dope is pushed through the spinneret and the solvent is evaporated (Clemons, 2016). Independently of the spinning method, a high molecular alignment is induced in the drawing step, which is key to the high stiffness and strength of the fibers (Clemons, 2016).

Several parameters such as the spinning speed, inner diameter and length of needle, and drying temperature, affect the alignment of CNF and hydrogen bond formation between CFNs (Kim et al., 2019). In the first report of CNF filament production through a wet-spinning process, the influence of spinning rates (from 0.1 to 100 m/min) were studied. The highest speed rate resulted in filaments with better mechanical properties (Iwamoto et al., 2011). By using post drawing, more efficient alignment of the nanofibrils is achieved resulting in further improvement of the mechanical properties of the fibers (Walther et al., 2011). A wet-stretching process is used to induce high fibers orientation, improving their Young’s Modulus from 8.2 to 33.7 GPa (Torres-Rendon et al., 2014). Various concentrations of CNF were tested, from 1 to 12%, being the higher ones processed through dry-spinning and the lowers by wet-spinning (Iwamoto et al., 2011; Hooshmand et al., 2015; Kim et al., 2019). In a study using a wet-spinning system 2% (m/m) CNF was considered the perfect solids content for achieving a high CNF alignment and filament strength, specifically, a Young Modulus of 37.5 GPa and Tensile Strength of 543.1 MPa (Kim et al., 2019). The mechanical properties of CNF filaments produced through the various processes are summarized in Table 2.

**TABLE 2:** Mechanical properties of different cellulose nanofiber filaments.

Spinning	Young’s Modulus (GPa)	Tensile Strength (MPa)	Elongation (%)	References
Wet-spinning	23.6	321	2.2	Iwamoto et al., 2011
	22.5	275	–	Walther et al., 2011
	33.7	289	1.6	Torres-Rendon et al., 2014
	28.9	369.6	–	Geng et al., 2017
	23.9	383.3	6.6	Kafy et al., 2017
	37.5	543.1	3.7	Kim et al., 2019
Dry-spinning	12.6	222	3.6	Hooshmand et al., 2015
	6.5	100	–	Ghasemi et al., 2017
Flow focusing	86	1570	–	Mittal et al., 2018
BNC wet-spinning	16.4	248.6	3.8	Yao et al., 2017
BNC stretching	65.7	826	2.5	Wang et al., 2017

**FIGURE 11:**
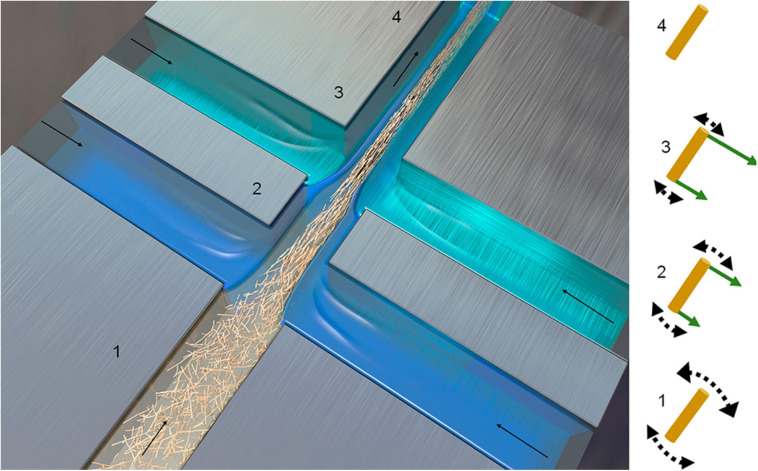
Schematic of double flow-focusing channel used for CNF assembly (Mittal et al., 2018).

Flow focusing is another method for CNF filaments production, resulting in fibers with the strongest tensile performance, as compared to other methods (Table 2). The fibers are generated by aligning a CNF suspension in a double flow focusing channel through coagulation with acid, as shown in Figure 11 (Mittal et al., 2018). The obtained filament has a Young’s modulus of 86 GPa and tensile strength of 1570 MPa, superior to those known natural or synthetic fibers (Mittal et al., 2018). The specific strength of this CNF fibers also exceeds that of metals, alloys, and glass fibers.

To our knowledge, the first and only company, to produce fibers without solvents, cellulose dissolution or any other harmful and complex chemical processes, is Spinnova Ltd. (Finland). The raw material is pulp from FSC certified wood. After mechanical pulping, the ground pulp passes through a single nozzle, where the fibers and fibrils rotate and align with the flow, providing a strong and elastic fiber network. The fiber is then spun and dried, suitable for spinning into yarn and then knitting or weaving into fabric (Salmela et al., 2016). The future goal is the recycling of the fibers for several times, minimizing the use of virgin materials. Spinnova fiber is now (2020) close to commercialization. The technology has been scaled up from a small pilot scale to an industrial pilot scale (Spinnova, 2020)^5^.

Filaments based on aligned BNC nanofibers were prepared by wet-spinning and drawing procedures (Yao et al., 2017). This macrofibers showed Young’s modulus of 16.4 GPa and tensile strength of 248.6 MPa using optimal process conditions (BNC 5.4 wt%; spinning rate 18.9 m/min), in which nanofibers displayed a high degree of alignment. The nanofiber suspension was prepared by TEMPO oxidation and then spun into an acetone coagulation bath (Yao et al., 2017). BNC Filaments without TEMPO oxidation were also produced. A super-strong and super-stiff cellulose macrofibers was obtained from aligned ultralong BNC nanofibers via a facile and scalable wet-drawing and wet-twisting method (Wang et al., 2017). The macrofibers showed perfect integrity and well aligned structure, with a tensile strength of 826 MPa and Young’s modulus of 65.7 GPa. BNC membranes were cut with a width of 7 mm and were wet-draw, becoming longer and thinner. From these, BNC macrofibers with diameters of around 300 μm were fabricated by wet-twisting and subsequently drying at 90°C for 12 h (Wang et al., 2017).

## Sustainability and Life Cycle Assessment

The rapid population growth and careless consumption of natural resources are causing serious global problems, such as air and water pollution and global warming. Fossil fuels exceeds 50% of the word’s total energy sources and based on present water consumption, water resources are likely to decrease by 30% in 2050 (Kazan et al., 2020). In the last few decades, the environmental problems caused by humankind are reaching dangerous levels, being the search and adoption of more environmentally sustainable processes a mandatory paradigm change.

In this regard, environmental impact assessment gains a significant importance to rate the environmental effects of industrial activities. LCA can be used to evaluate a product, process or activity repercussions on the environment (Van Der Velden et al., 2014). “Cradle to gate” LCA studies take in account the raw materials and fuels used, as well all the processes involved until the product is delivered at the factory gate for further processing; “cradle to grave” involves, in addition to the later, post-manufacturing processes until the product (garment) end of life (Dibdiakova and Timmermann, 2014).

**FIGURE 12:**
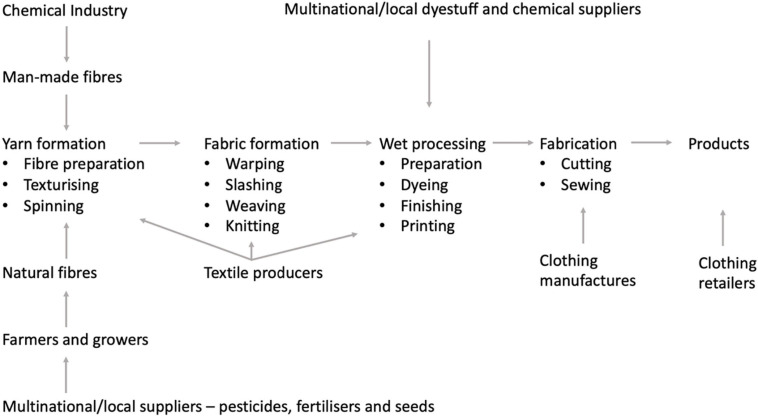
The business involved in the textile and clothing supply chain [adapted from Choudhury (2014)].

The global textile supply chain is complex, involving several stages (Figure 12). It is widely recognized that the textile industry is a major contributor to the environmental pollution and resource consumption. Among all, this industry is placed at fifth place in terms of the release of chemical oxygen demand (COD), implying large quantities of wastewater production and chemical consumption (Roos et al., 2018; Zhang Y. et al., 2018). Along COD, textile wastewaters may contain substances with a high biological oxygen demand (BOD), total suspended solids, oil and grease, sulfides, sulfates, phosphates, chromium, copper, and/or the salts of other heavy metals. The major part of the chemical substances used in textile manufacture are generated during wet processing (dyeing, washing, printing, and fabric finishing). Textile dyeing and finishing mills utilize 200 tons of water for every metric ton of textiles produced. The textile industry is a major energy-consuming industry with low efficiency in energy utilization. A large quantity of non-renewable energy sources is consumed in the form of electricity, not so much in the process of textile production (15–20%) but mostly in subsequent laundering processes during consumer use (75–80%) (Choudhury, 2014).

Although the entire process of textile production generates hazardous wastes, in this review we focus only on raw materials and fibers production. A cradle-to-gate LCA study, shown in Table 3, makes possible to evaluate the environmental impact of different fiber production processes (Dibdiakova and Timmermann, 2014). It is important to mention that, for a true comparative LCA, the same framework must be used for all stages of the life-cycle of a commercial product, process, or service (this means using the same criteria for a life cycle inventory of the required resources (energy and materials) across the value chain and determining the corresponding emissions to the environment). Data here collected was obtained from different sources and therefore a direct comparison of the impact categories is not straightforward.

**TABLE 3:** LCA cradle-to-gate of different impact categories per 1000 kg of fiber (functional unit) produced under different methods.

Impact Category	Cotton Fiber	Tencel (lyocell)	Viscose Austria	Viscose Asia	Polyester
Water Use (m^3^)	5730 ^1^	263 ^2^	445 ^2^	319 ^3^	125 ^3^
Energy Demand (GJ)	55 ^1^	65 ^3^	70 ^3^	106 ^3^	96 ^3^
Global Warming (kg CO_2_-Equiv.)	2000 ^1^	50 ^3^	−250 ^3^	3800 ^3^	4100 ^3^
Acidification (kg SO_2_-Equiv.)	41 ^1^	13 ^3^	14 ^3^	45 ^3^	21 ^3^
Eutrophication (kg phosphate-Equiv.)	22 ^1^	1.9 ^3^	1.2 ^3^	2.3 ^3^	1.2 ^3^
Ozone Depletion (×10^–3^ kg CFC11 Equiv.)	0.2 ^1^	0.07 ^3^	0.03 ^3^	0.28 ^3^	0.07 ^3^
Land Use (ha/year)	1.07 ^1^	0.22 ^3^	0.69 ^2^	0.35 ^3^	0 ^4^

*^1^Cotton Incorporated (2012). ^2^Taylor (2010). ^3^Shen and Patel (2010). ^4^Dibdiakova and Timmermann (2014).*

Polyester, the most widely used synthetic fiber, has a lower water consumption than the other cellulose fibers, but has the highest impact on global warming from CO_2_ emissions and also a high demand on energy (Zambrano et al., 2019). Cotton is the most used natural raw material in textile industry (Kazan et al., 2020). Nutrients and pesticides are used in raw material production to increase quantity and product quality and so, they pollute the groundwater and surface water. Up to 25% of global pesticide usage in agriculture corresponds to cotton production (Kazan et al., 2020). Although cotton has the advantage of being biodegradable, it has a significant water consumption, with an average global water footprint of 5730 m^3^/ton in crops production and required water for processing (Cotton Incorporated, 2012). Some of the negative aspects of cotton production can be overcome by growing organic crops but it still has many negative environmental aspects compared to other fibers (Textile Exchanges, 2014; La Rosa and Grammatikos, 2019).

Regarding the processing stage, the Viscose process has different environmental impacts depending on where it is produced (Shen et al., 2010). The problems with these fibers are the chemicals used in the regeneration process and the need for large plantation areas for wood production. These plantation areas need to have a sustainable managing system, and preferably be certified, and, in the past, this was not always true.

Currently, the most environmentally friendly fibers on the market are produced by the Lyocell process (Shen et al., 2010). The solvent used is non-toxic and recyclable being the main challenge the sustainable sourcing of cellulose. Tencel, made of cellulose from sustainable eucalyptus plantations, which grows quickly and requires no irrigation or pesticides, is the best option on the market regarding its environmental impact. BNC may be the solution to Lyocell flaws as it can be produced anywhere without the use of forest resources. An attributional LCA was applied to a projected production of BNC, by static culture, following a cradle-to-gate approach which comprises the removal of natural resources and their conversion, the production of BNC, the utilities, the energy and equipment used, as well as the treatment and disposal of the waste produced by the BNC process chain. The results showed that water was the main resource used, most of which being returned back to fresh water after treatment. The BNC manufacture facility itself contributed little extent to the consumption of resources and environmental impact of the global life cycle. The materials production were accountable for most of natural resources utilized (water), and the emissions liberated to the environment, in this case released to fresh water (Forte et al., 2019).

## Conclusion

Textile industry evolved over the years. Several types of fibers have been produced to fulfill people’s needs and demands, polyester, cotton and Viscose being the most used. However, these fibers have environmental implications associated to raw material processing and transport, filament manufacture, product and byproducts disposal. Currently, the most environmentally friendly cellulose fibers on the market are produced by the Lyocell process. Current trends include the development of filaments without using solvents, the development of new solvents and new sources of fibers, which include different nanocelluloses, namely bacterial cellulose. This presents a superior outlook in terms of sustainability. However, the large scale production of this biotech cellulose is still to be demonstrated. Further research is still needed to scale up its production as their production cost.

Man-made cellulosic fibers are here to stay. Since its discovery, more than 100 years ago, they assume an important role in the textile industry. Along the history its commercialization faced one major problem: the price. These fibers are more expensive than cotton, and a lot more expensive than synthetics. But the world has changed. The population growth, and the increasing economic power in some underdeveloped economies are putting a tremendous pressure under some industries, especially those producing essential goods, like the textile industry for garment. To allow an increase in textile production, fibers production must increase also. Cotton cannot respond to this increase, because there is no arable land available, and nor the synthetics, presenting some major problems on recyclability, biodegradability, row material availability in the future and microplastics pollution. MMCF will have a very important role in the response of this need. And in the eternal quest for optimization, the MMCF will evolve along the entire value chain to minimize its environmental foot print, improving production methods and raw materials supply.

## Author Contributions

MG and FD: conceptualization. CF: writing. CF and CG: data analysis. CF, NA, CG, MG, and FD: writing-review and editing. All authors approved the manuscript.

## Conflict of Interest

The authors declare that the research was conducted in the absence of any commercial or financial relationships that could be construed as a potential conflict of interest.

## Footnotes

1 https://unfashionalliance.org/

2 https://emergingtextiles.com/

3 https://nanollose.com/

4 https://ioncell.fi/commercialization/

5 https://spinnova.com/our-method/fibre/
